# Phytotoxicity of Silver Nanoparticles on Tobacco Plants: Evaluation of Coating Effects on Photosynthetic Performance and Chloroplast Ultrastructure

**DOI:** 10.3390/nano11030744

**Published:** 2021-03-16

**Authors:** Petra Peharec Štefanić, Karla Košpić, Daniel Mark Lyons, Lara Jurković, Biljana Balen, Mirta Tkalec

**Affiliations:** 1 Department of Biology, Faculty of Science, University of Zagreb, Horvatovac 102a, HR-10000 Zagreb, Croatia; ppeharec@biol.pmf.hr (P.P.Š.); karla.kospic@biol.pmf.hr (K.K.); bbalen@biol.pmf.hr (B.B.); 2 Center for Marine Research, Ruđer Bošković Institute, G. Paliaga 5, 52210 Rovinj, Croatia; Daniel.Mark.Lyons@irb.hr (D.M.L.); Lara.Jurkovic@irb.hr (L.J.)

**Keywords:** chlorophyll fluorescence, chloroplast ultrastructure, *Nicotiana tabacum* L., photosynthetic pigments, silver ions, silver nanoparticles

## Abstract

Silver nanoparticles (AgNPs) are the most exploited nanomaterial in agriculture and food production, and their release into the environment raises concern about their impact on plants. Since AgNPs are prone to biotransformation, various surface coatings are used to enhance their stability, which may modulate AgNP-imposed toxic effects. In this study, the impact of AgNPs stabilized with different coatings (citrate, polyvinylpyrrolidone (PVP), and cetyltrimethylammonium bromide (CTAB)) and AgNO_3_ on photosynthesis of tobacco plants as well as AgNP stability in exposure medium have been investigated. Obtained results revealed that AgNP-citrate induced the least effects on chlorophyll *a* fluorescence parameters and pigment content, which could be ascribed to their fast agglomeration in the exposure medium and consequently weak uptake. The impact of AgNP-PVP and AgNP-CTAB was more severe, inducing a deterioration of photosynthetic activity along with reduced pigment content and alterations in chloroplast ultrastructure, which could be correlated to their higher stability, elevated Ag accumulation, and surface charge. In conclusion, intrinsic properties of AgNP coatings affect their stability and bioavailability in the biological medium, thereby indirectly contributing changes in the photosynthetic apparatus. Moreover, AgNP treatments exhibited more severe inhibitory effects compared to AgNO_3_, which indicates that the impact on photosynthesis is dependent on the form of Ag.

## 1. Introduction

The rapidly growing production and utilization of engineered nanomaterials (ENMs) in industry and various consumer products has led to rising environmental health concerns [1,2]. Among various types of ENMs, nanoparticles (NPs) have found numerous applications, especially in agriculture, biomedical engineering, and environmental remediation techniques [3] due to their unique properties, such as high surface-to-volume ratio and, consequently, reactivity greater than that of macrosized particles [4]. However, there is a complex association between the physicochemical properties at the nanoscale and the biological activity of NPs that may potentially give rise to toxicity [5,6,7]. Due to their well-known antimicrobial properties, silver NPs (AgNPs) are of particular interest in a broad range of medical applications [8,9], and are the most exploited nanomaterial in agriculture and food production [10]. However, increasing usage and unregulated release of AgNPs into aquatic and terrestrial systems gives cause for concern about their impact on the environment and living organisms, among which plants, as sessile organisms, are particularly affected. Moreover, since it has been shown that AgNPs are able to enter plant cells [11,12], plants can serve as an entry point for transport and biodistribution of AgNPs in the food chain [13]. AgNPs can impose both positive [14,15] and negative impacts [16,17] on plant growth and metabolism, which is found to be dependent on various factors, including the plant species, age, and tissue type [13]. Furthermore, the characteristics of AgNPs such as size, concentration, and charge [18,19] as well as the experimental conditions including duration and method of exposure [20,21,22] also play significant roles, influencing the stability of AgNPs and their susceptibility to transformation [19,23]. In recent studies, it was revealed that AgNP stability is also dependent on the composition and type of the exposure medium [24,25]. The use of various surface coatings in their synthesis influences the physiochemical properties of AgNPs, not only by reducing particle agglomeration and enhancing their stability but also by modulating toxic effects [4,18,26,27]. The synthesis method of AgNPs can also have an impact on their toxicity [28]. Biological synthesis of AgNPs is increasingly used because it is cheaper and less harmful compared to physical and chemical synthesis [29], and recent findings show that biologically synthesized AgNPs are less toxic [28]. Once absorbed by plants, AgNPs may induce toxicity by generation of reactive oxygen species (ROS) leading to induction of oxidative stress [30], disruption of cell membrane integrity [31], and damage to biologically important macromolecules (DNA and proteins) [13], thus impairing plant growth and development.

Photosynthesis is one of the most important biological processes, providing energy as well as oxygen for life on our planet [32]. Previous studies have reported the impact of AgNPs on photosynthesis through several mechanisms, among which the change in content of photosynthetic pigments is the most notable. Namely, chlorophyll, as a major photosynthetic pigment, is responsible for capturing light energy and its content can be used as an indicator of plant photosynthetic capacity [33]. A decrease in chlorophyll content has been reported in higher plants such as mustard [34], pea [35], and rice [36] upon exposure to AgNPs. Furthermore, several studies have also reported a significant reduction of carotenoids [37,38]. On the contrary, foliar application of AgNPs to lettuce failed to show any toxic effects on photosynthetic pigment content [39], while in fenugreek, it increased the content of all photosynthetic pigments [40].

Apart from the content of photosynthetic pigments, measurement of chlorophyll *a* fluorescence has been proposed as a sensitive and efficient method for detecting the impacts of environmental stress on photosynthetic efficiency [41]. Among different fluorescence parameters, the most frequently measured is the quantum yield (*F_v_*/*F_m_*) of photosystem II (PSII), corresponding to the efficiency by which an absorbed photon will be trapped by PSII reaction centers [42], and which was found to be significantly affected upon exposure to AgNPs [13]. A decrease in PSII efficiency was reported in AgNP-treated aquatic plants *Spirodela polyrhiza* [43] and *Lemna gibba* [44], where measurements of chlorophyll *a* fluorescence showed strong inhibitory effects of AgNPs on energy transfer from light harvesting complexes to reaction centers, the deterioration of the PSII water splitting system, and the inactivation of PSII reaction centers. Moreover, it was found that AgNPs significantly disrupt photosynthesis in terrestrial plants, where significant decreases in electron transfer and the rate of photosynthesis were observed [34,35,45]. Significantly lower values of relative electron transport rate and coefficient of photochemical quenching were also obtained in tobacco seedlings exposed to AgNPs [25]. However, there are also reports that treatments with AgNPs did not induce any significant change in PSII efficiency [46,47]. This divergence of effects on photosynthesis can be attributed to differences in plant species, type, and time of exposure as well as to the use of AgNPs with different physicochemical characteristics [13].

Several studies observed AgNP impact on morphology and ultrastructure of leaves of exposed plants, where the most notable changes were reported for chloroplast ultrastructure, including disturbances in their shape, thylakoid system, plastoglobules, and starch content [13]. The most significant modifications of chloroplast ultrastructure reported upon exposure of plant to AgNPs included swollen and ruptured chloroplasts with an increased number of plastoglobules [11], a dilated thylakoid system [12], and thinner grana lamellae [48].

The aim of this study was to investigate biochemical and ultrastructural changes in the photosynthetic apparatus of tobacco plants (*Nicotiana tabacum* L.) upon exposure to three differently coated AgNPs, which have been laboratory-synthesized and characterized, to elucidate how the intrinsic properties of coatings affect AgNPs stability and bioavailability, and thus indirectly contribute to alterations in photosynthetic apparatus. To our knowledge, the only study investigating the toxicity of differently coated AgNPs in photosynthesis was assessed in freshwater algae *Chlamydomonas reinhardtii* [26], whereas data for terrestrial higher plant species are missing. As the object of this study, tobacco was chosen, as it is an economically important plant and a frequently used model organism in abiotic stress research [49,50], particularly as it is considered relatively tolerant to environmental stress [51].

## 2. Materials and Methods

### 2.1. AgNPs Synthesis

All chemicals were purchased from Sigma-Aldrich, St. Louis, MO, USA and were at least of analytical purity, and ultrapure water (Milli-Q, 18.2 MΩ∙cm, Merck Millipore, Billerica, MA, USA) was used in all syntheses.

Synthesis of citrate-coated AgNPs was carried out by addition of 5 mL aqueous sodium citrate solution (1% *w*/*v*) to 120 mL of a stirred boiling aqueous solution of AgNO_3_ (99.999% purity, 0.02 g). Upon change of color from transparent to pale yellow, the solution was cooled to room temperature under a stream of cold water and stored until subsequent characterization.

Syntheses of AgNPs coated with polyvinylpyrrolidone (AgNP-PVP) and cetyltrimethylammonium bromide (AgNP-CTAB) were performed as reported by Biba et al. [19] with minor modifications. Briefly, similar to citrate-capped AgNPs, synthesis of PVP-coated AgNP was carried out by adding 5 mL of a 1% (*w*/*v*) sodium citrate solution to 120 mL ultrapure water to which PVP (0.019 g, average molecular weight 40 × 10^3^ g mol^−1^) and AgNO_3_ (0.02 g) had been previously added. The solution was held at boiling point until the color changed to pale yellow-orange, at which point it was cooled to room temperature under running cold water.

AgNP-CTAB was synthesized by adding 65 mL ultrapure water containing 0.0043 g CTAB and 0.02 g AgNO_3_ by burette in a slow but constant stream to a stirred 60 mL aqueous solution containing 0.01 g ascorbic acid. Both solutions had been cooled to 0 °C before mixing. A color change from transparent to pale orange indicated completion of silver reduction, after which the solution was stored at 4 °C until analysis.

### 2.2. Characterization of AgNP Stock Solutions

Analyses of physical and chemical characteristics of citrate-, PVP-, and CTAB-coated AgNP stock solutions in ultrapure water were mainly performed as reported by Peharec Štefanić et al. [12] and Biba et al. [19]. AgNP formation was confirmed by the presence of a surface plasmon resonance (SPR) peak using a UV-Vis spectrophotometer (Unicam, Cheshire, UK). The AgNP hydrodynamic diameters were evaluated using dynamic light scattering (DLS), whereas their ζ potential was determined by measuring electrophoretic light scattering (ELS), with a Zetasizer Nano ZS (Malvern Panalytical, Malvern, UK) equipped with a green laser (532 nm). Intensity of scattered light was detected at the angle of 173°. For data processing, Zetasizer software version 6.32 (Malvern Panalytical, Malvern, UK) was used. The AgNP hydrodynamic diameters are given as the average value of 10 measurements (mean ± S.D., *n* = 10) and are reported as the volume size distributions, whereas AgNP ζ potentials are reported as the average of five measurements (mean ± S.D., *n* = 5).

The concentration of Ag^+^ deriving from dissolution of AgNP in ultrapure water was determined by centrifugal ultrafiltration (Millipore Amicon Ultra-4 3K, Merck Millipore, Billerica, MA, USA) through a membrane with a 3 kDa molecular weight cutoff. Suspensions were centrifuged for 30 min at 15,000× *g*. Total Ag concentration in the AgNP dispersions and the filtrates (Ag^+^) (*n* = 5) were determined in acidified solutions (10% *v*/*v* HNO_3_) using an ELAN DRC-e (Perkin Elmer, Waltham, MA, USA) inductively coupled plasma mass spectrometer (ICP-MS). Ag concentration was calculated according to a calibration curve obtained with a set of standards of known concentrations. Detection limit and limit of quantification (LOQ) were 0.2 and 1 mg kg^−1^, respectively.

AgNP visualization was carried out by placing 2 μL of each AgNP stock solution onto Formvar^®^/carbon copper grids, which were air-dried and subsequently examined using a monochromated TF20 (FEI Tecnai G2, FEI, Hillsboro, OR, USA) transmission electron microscope (TEM), with a Schottky cathode and operating at 200 kV. The TEM was equipped for energy-dispersive X-ray spectroscopy (EDX) with a SiLi detector and an ultrathin window. For each stock solution, four replicas (*n* = 4) were analyzed.

### 2.3. Plant Material and Exposure Experiments

Tobacco (*Nicotiana tabacum* L. cv Burley) plants were cultivated in in vitro conditions as previously described [49,50]. Seeds were surface-sterilized with 50% (*v*/*v*) sodium hypochlorite and subsequently washed with deionized H_2_O. Solid Murashige and Skoog [52] nutrient medium supplemented with 500 mg L^−1^ MES (2-(N-morpholino)ethanesulfonic acid), 1.5 g L^−1^ sucrose, and 2.2 g L^−1^ Phytagel (pH 5.6) [53] was used for seed germination and plant growth. In each Erlenmeyer flask (300 mL), two tobacco seeds were placed on the surface of the culture medium (50 mL) and left to germinate and grow for two months in the growth chamber (16/8 light/dark cycle, light intensity 90 µmol m^−2^ s^−1^, and temperature 24 °C) until adult plants with a well-developed root system and shoots with differentiated leaves were obtained [11].

For exposure experiments, adult plants of similar sizes with 2–3 leaves were transferred to liquid ½ strength MS medium supplemented with 25, 50, and 100 μM AgNP-citrate, AgNP-PVP, AgNP-CTAB, or AgNO_3_. Control plants were cultured in liquid ½ strength MS medium without silver. Control and treated plants were grown for 7 days in the growth chamber under the aforementioned conditions. The experiment was performed two times with three replicates for each treatment.

### 2.4. AgNP Stability in a ½ Strength MS Medium

The stability of 100 µM AgNP-citrate, AgNP-PVP, and AgNP-CTAB solution in a liquid ½ strength MS medium was evaluated with UV-Vis absorption spectra, measuring their hydrodynamic diameters (DLS) and ζ potentials (ELS) using Zetasizer Nano ZS (Malvern Panalytical, Malvern, UK) and TEM analyses as described previously in Section 2.2. All measurements were conducted at 25 °C. Measurements were performed at 0 and 10 min as well as 1, 4, 24, and 48 h after addition of AgNPs to the nutrient medium. Hydrodynamic diameters are reported as an average value of 10 measurements and the size distributions are reported as volume distributions. The ζ potentials of AgNPs are reported as average values of six measurements.

### 2.5. Determination of Ag Content

Measurement of Ag content in treated plant material was performed as previously reported by Cvjetko et al. [11]. Briefly, leaves of exposed as well as control tobacco plants were removed from the shoot and dried in a microwave oven for 24 h at 80 °C, after which they were powdered using a mortar and pestle. Tissue was digested in a microwave oven (ETHOS SEL Milestone, Shelton, CT, USA) according to the EPA 3051a method—first in 10 mL of concentrated HNO_3_ at 130 °C for 10 min, then at 180 °C for another 15 min. The second step was digestion in 1 mL of H_2_O_2_ at 85 °C for 5 min and then at 130 °C for 4 min. The samples were cooled and subsequently diluted with 1% (*v*/*v*) HNO_3_ up to a final volume of 50 mL. ELAN DRC-e ICP-MS (Perkin Elmer, Waltham, MA, USA) was used for determination of the total Ag content. To calculate the Ag concentration, a calibration curve obtained with a set of standards of known concentrations was used. The detection limit and limit of quantification (LOQ) were 0.05 and 0.1 mg kg^−1^, respectively. Spike recovery tests were 95.6% for leaves of AgNO_3_-treated plants, and 95.2%, 95.4%, and 94.9% for leaves of plants exposed to AgNP-citrate, AgNP-PVP, and AgNP-CTAB, respectively.

### 2.6. Chlorophyll a Fluorescence

In dark-adapted (30 min) leaves of both treated and control tobacco plants, minimal fluorescence (*F_0_*) was determined with a FluorPen FP100 (Photon Systems Instruments, Brno, Czech Republic). After a short pulse of saturating light (3000 µmol photons m^−2^ s^−1^), maximum fluorescence (*F_m_*) was measured. To measure steady-state fluorescence (*F*) and maximum fluorescence (*F’_m_*) in a light-adapted state, the leaf was illuminated with actinic light of 100 µmol photons m^−2^ s^−1^. Maximum photochemical quantum yield of PSII (*F_v_*/*F_m_*), effective quantum yield of PSII (*Φ_PSII_*), nonphotochemical quenching (*NPQ*), and coefficient of photochemical quenching (*qP*) were calculated according to Maxwell and Johnson [54].

### 2.7. HPLC Analysis of Photosynthetic Pigments

Freeze-dried leaf samples were homogenized in 96% cold acetone with 0.3 mg mL^−1^ calcium carbonate (CaCO_3_) in a dim light. The pigments were separated by high-performance liquid chromatography (HPLC, Perkin Elmer, Waltham, MA, USA) with a diode array detector (DAD) and non-endcapped Zorbax ODS column (4.6 × 250 mm, 5 µm particle size, Agilent Technologies, Santa Clara, CA, USA). For pigment elution, the method described by Thayer and Björkman [55] was used with slight modification—100% solvent A (acetonitrile:methanol:water, 84:12:4) for the first 2 min followed by a 14 min linear gradient to 100% solvent B (methanol:ethyl acetate, 68:32), which continued isocratically for the next 9 min, with a flow rate of 1 mL min^−1^. The pigments neoxanthin, violaxanthin, antheraxanthin, zeaxanthin, lutein, β-carotene, chlorophyll *a*, and chlorophyll *b* were detected by absorbance at 440 nm and quantified against known standards (DHI Water and Environment, Hørsholm, Denmark).

### 2.8. Microscopy Analyses

For localization of AgNPs and ultrastructural analyses of leaves of control plants and plants exposed to 100 μM AgNPs coated with citrate, PVP, and CTAB as well as to 100 μM AgNO_3_, small pieces of leaf tissue were fixed with 1% (*w*/*v*) glutaraldehyde in 50 mM cacodylate buffer (pH 7.2) for 1 h at +4 °C. Subsequently, they were washed twice with cold 50 mM cacodylate buffer (pH 7.2) and postfixed with 1% (*w*/*v*) osmium tetroxide in the same buffer for 1 h at + 4 °C, followed by a 10-min wash in ice-cold water. After dehydration in a graded series of ethanol, the tissue was embedded in Spurr’s resin.

Semithin sections of fixed material were stained with a mixture of 2% (*w*/*v*) toluidine blue and 2% (*w*/*v*) borax and examined using a light microscope. Ultrathin sections were stained with 2% (*w*/*v*) uranyl acetate and 2% (*w*/*v*) lead citrate and examined using an FEI Morgagni 268D electron microscope (FEI, Eindhoven, The Netherland) operated at 70 kV for ultrastructural study and monochromated TF20 (FEI Tecnai G2, FEI, Hillsboro, OR, USA) TEM for confirmation of AgNP localization in the tobacco cells.

### 2.9. Statistical Analysis

The data were analyzed using one-way ANOVA followed by the least significant difference (LSD) test using the STATISTICA 13.0 (TIBCO Software Inc., Palo Alto, CA, USA) software package. Differences between means were considered statistically significant at *p* ≤ 0.05.

## 3. Results

### 3.1. AgNP Characterization

**Figure 1:**
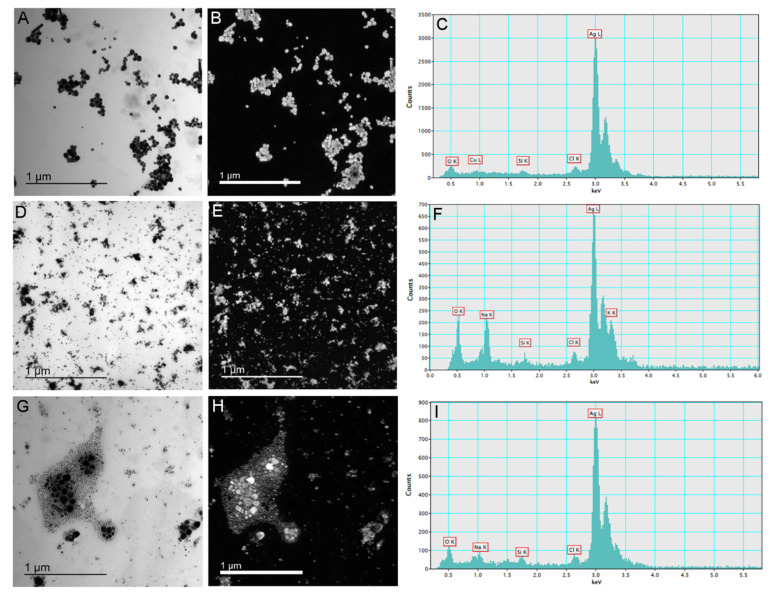
Stock solutions of silver nanoparticles (AgNPs) coated with citrate (AgNP-citrate), polyvinylpyrrolidone (PVP; AgNP-PVP), and cetyltrimethylammonium bromide (CTAB; AgNP-CTAB) in ultrapure water investigated by transmission electron microscopy. Micrographs (**A**,**D**,**G**)—bright field image; (**B**,**E**,**H**)—silver elemental map; and energy-dispersive X-ray spectra—(**C**,**F**,**I**) for AgNP-citrate, AgNP-PVP and AgNP-CTAB, respectively. For each stock solution, four replicates (*n* = 4) were analyzed.

Synthesized AgNP-citrate, AgNP-PVP, and AgNP-CTAB were characterized using UV-Vis spectroscopy, TEM, DLS, and ELS, and the results are presented in Figure 1 and Table 1. TEM micrographs showed that AgNPs generally had a spherical shape with a small number of rodlike particles present (Figure 1A,D,G), whereas energy dispersive X-ray analysis indicated that all the imaged particles were comprised of silver (Figure 1C,F,I). UV-Vis spectra showed SPR peaks of AgNP-citrate, AgNP-PVP, and AgNP-CTAB at 420, 465, and 410 nm, respectively, confirming the synthesis of nanosized silver particle dispersions. These SPR absorption peak maxima are consistent with silver nanoparticles of 50, 80, and 40 nm nominal diameters, respectively, and the quantity of ionic silver in the as-synthesized dispersions was ≤0.5% (Table 1).

The volume size distributions determined by DLS indicated hydrodynamic diameters of about 24 nm, 58 nm, and 56 nm for AgNP-citrate, AgNP-PVP, and AgNP-CTAB, respectively. A population of particles of about 6 nm diameter was found for AgNP-citrate which may be related to small silver clusters that might have formed early during synthesis. A peak corresponding to a hydrodynamic diameter of about 28 nm was also noted for AgNP-CTAB and ascribed to small particles arising at the beginning of the reaction from rapid reduction of silver when the ratio of reducing agent to silver was high. Small agglomerates were evidenced by a population with hydrodynamic diameters of about 163 nm. The zeta (ζ) potential values were found to be about −24 mV for AgNP-citrate and 45 mV for AgNP-CTAB, while AgNP-PVP showed an expected ζ potential close to neutral (−4 mV). Size distribution obtained from TEM showed that AgNP-citrate nanoparticles were mostly around 30 to 60 nm in size. AgNP-PVP nanoparticles were mostly between 20 and 50 nm, whereas AgNP-CTAB nanoparticles were in the range from 15 nm to 30 nm with a small portion of bigger ones (70–80 nm) (Figure S1).

### 3.2. AgNP Stability in Liquid Medium

The dispersion system employed in this study was liquid ½ strength MS medium. Although the investigated AgNPs had different coating ligands, they were all prone to rapid agglomeration after exposure to the nutrient medium.

**Figure 2:**
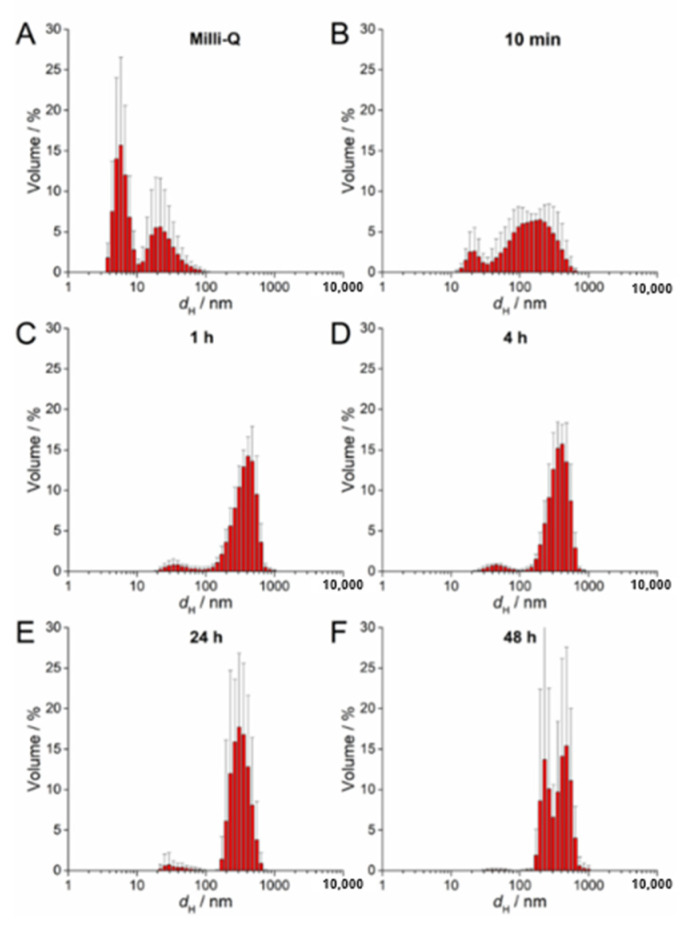
Size distribution plots of 100 µM AgNP-citrate in (**A**) ultrapure water and after (**B**) 10 min, (**C**) 1 h, (**D**) 4 h, (**E**) 24 h, and (**F**) 48 h in exposure solution (liquid ½ strength MS medium).

**Figure 3:**
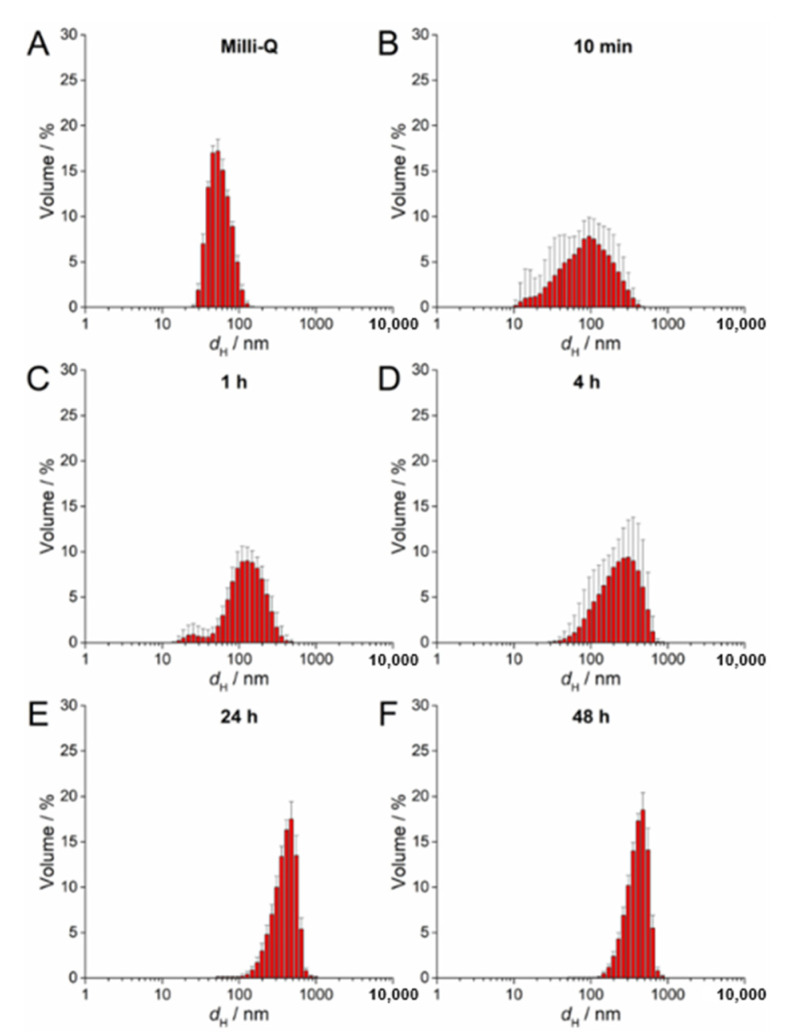
Size distribution plots of 100 µM AgNP-PVP in (**A**) ultrapure water and after (**B**) 10 min, (**C**) 1 h, (**D**) 4 h, (**E**) 24 h, and (**F**) 48 h in exposure solution (liquid ½ strength MS medium).

UV-Vis absorption data for 100 µM AgNP-citrate in ½ MS medium showed a slight shift of the surface plasmon resonance (SPR) toward higher wavelength and an increase in intensity up to 4 h after addition of nanoparticles to the medium (Figure S2A). This may indicate some reduction of ionic silver and growth in particle size. In parallel, the SPR became broader, suggesting that agglomeration of the particles was occurring in parallel, which was additionally confirmed by TEM analysis (Figure S2A). DLS measurements confirmed agglomeration by showing a shift in volume size distribution to larger hydrodynamic diameters 10 min after nanoparticle addition to the medium (about 213 nm; Figure 2B; Table 2) compared to ultrapure water (Figure 2A). Agglomerates attained an average diameter of about 422 nm after 1 h, with the diameter of the agglomerates gradually decreasing to about 245 nm after 48 h (Figure 2C–F; Table 2). The ζ potential remained relatively constant over 48 h and varied in the range −23.78 to −19.78 mV, suggesting that the colloid retained some stability, which is consistent with DLS data where agglomerates remained relatively small, as well as a small population of nanoparticles of 30–40 nm diameter persisting, over the same period (Table 2). Similar to AgNP-citrate, the SPR of AgNP-PVP increased in intensity in the period 1–4 h after addition of nanoparticles to the medium, and then slowly decreased (Figure S2B). Again, this may be related to reduction of residual Ag^+^ ions from the nanoparticle synthesis reaction followed by gradual oxidative dissolution of the nanoparticles. While there was little peak broadening with time, suggesting that significant agglomeration had not taken place, DLS data indicated that agglomeration was occurring, though at a slower rate than for AgNP-citrate, with agglomerate diameters of about 124 nm noted after 10 min and stabilizing at about 450 nm after 24 h (Figure 3A–F; Table 2). Agglomeration was additionally confirmed by TEM analysis (Figure S2B). Unlike AgNP-citrate, the ζ potential of AgNP-PVP became more negative after being added to the medium (from −4 mV to −9.6 mV), reflecting increased electrostatic repulsion between nanoparticles and consistent with the slower rate of agglomeration noted (Table 2).

**Figure 4:**
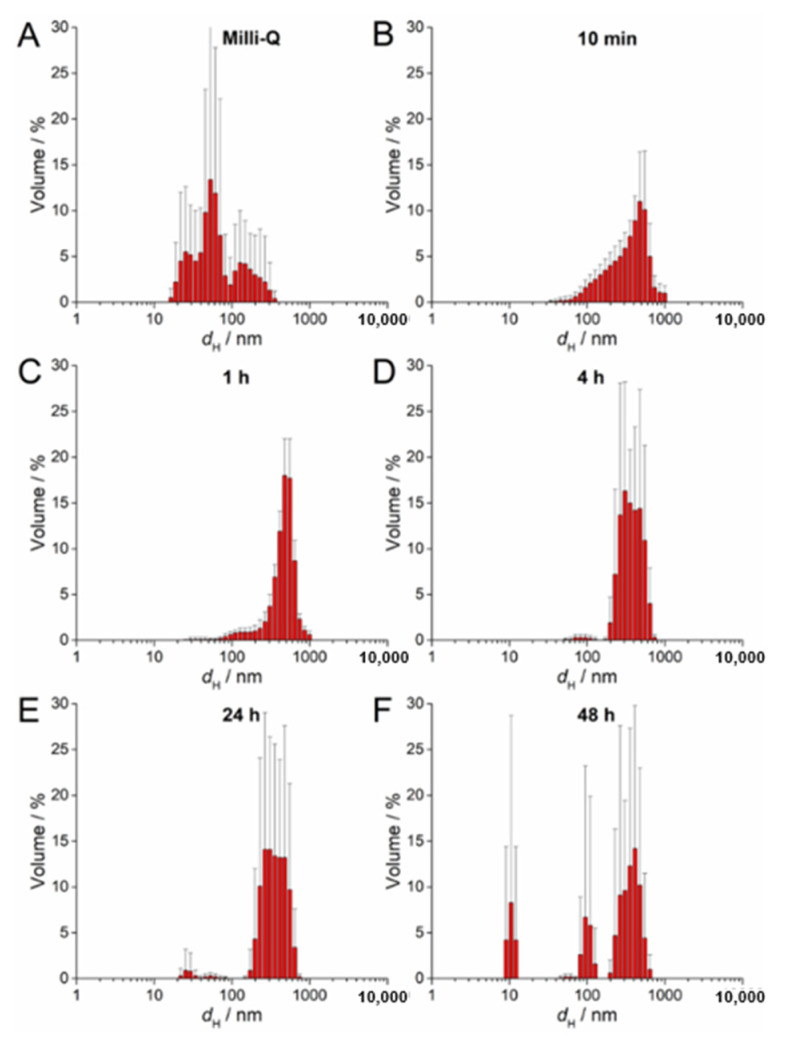
Size distribution plots of 100 µM AgNP-CTAB in (**A**) ultrapure Q water and after (**B**) 10 min, (**C**) 1 h, (**D**) 4 h, (**E**) 24 h, and (**F**) 48 h in exposure solution (liquid ½ strength MS medium).

The SPR of AgNP-CTAB showed different behavior compared to the citrate and PVP- coated AgNPs. There was no increase in absorbance over several hours but rather a constant decrease in intensity (Figure S2C). Peak broadening was noted almost immediately after the AgNP-CTAB was added to the liquid ½ strength MS medium, indicating rapid agglomeration, which was also observed with TEM (Figure S2C). DLS measurements of AgNP-CTAB revealed nanoparticles with a number of *d*_H_ values in ultrapure water (Figure 4A), although after exposure to liquid ½ strength MS medium these nanoparticles rapidly agglomerated such that, within 10 min, agglomerates of about 500 nm had already formed (Figure 4B–F; Table 2). Concurrently, the ζ potential rapidly decreased from 44.67 mV to 7.00 mV after 1 h, consistent with the dispersion losing stability and agglomeration occurring (Table 2). However, after 48 h, an additional AgNP-CTAB population of about 100 nm was recorded by DLS and accompanied by a slight increase in ζ potential, indicating some restabilization of small agglomerates by CTAB (Figure 4F; a second small population of about 10 nm may possibly be related to CTAB-derived micelles).

### 3.3. Ag Content

Ag in leaves of tobacco plants exposed to different AgNPs and AgNO_3_ treatments accumulated accordingly with the increasing concentrations of both treatments, with the highest (significant) values noted after exposure to 100 μM of AgNPs and AgNO_3_ (Table 3). However, after treatments with the lower concentrations (25 and 50 µM) of AgNP-citrate and the lowest concentration of AgNP-PVP and AgNO_3_ (25 µM), the increase of Ag content was slight and not significant compared to the control. Moreover, Ag uptake in leaves was lower after exposure to AgNP-citrate treatments compared to the corresponding AgNO_3_ treatments, whereas to the contrary, it was higher after exposure to AgNP-CTAB. In leaves of control plants, Ag content was below the instrument detection limit of 0.1 mg L^−1^.

### 3.4. Effects on Chlorophyll a Fluorescence 

Measurements of chlorophyll *a* fluorescence parameters are presented in Table 3. Maximum quantum yield of PSII (*F_v_*/*F_m_*) of plants treated with differently coated AgNPs or AgNO_3_ in concentrations of 25, 50, or 100 µM showed no difference compared to values of control plants. In general, there was no difference between plants treated with different concentrations of the same Ag treatment (either AgNPs or AgNO_3_). However, some exceptions were noted; *F_v_*/*F_m_* was lower in plants treated with the 25 µM AgNP-PVP compared to 50 and 100 µM treatments, whereas exposure to 50 µM AgNP-CTAB resulted in reduced values in comparison to the highest applied concentration (100 µM). The only difference among treatments with AgNPs with different coatings of the same concentration was noted for the 50 µM concentration, where *F_v_*/*F_m_* was lower in leaves of plants exposed to AgNP-CTAB compared to treatments with AgNP-PVP and AgNP-citrate.

Treatments with different concentrations of AgNP-citrate and AgNP-PVP as well as AgNO_3_ did not cause any significant change in effective quantum yield of PSII (*ΦPSII*) compared to control plants. However, in plants exposed to AgNP-CTAB, values were lower than the control ones, and significantly different from the control after treatments with 25 and 100 µM concentrations. Accordingly, these plants had the lowest values compared to the plants treated with corresponding concentrations of AgNP-citrate, AgNP-PVP and AgNO_3_. However, there was no difference in *ΦPSII* among different concentrations of AgNP-CTAB treatments. Plants exposed to AgNP-citrate, although having values similar to the control, showed reduced *ΦPSII* in comparison to plants treated with AgNO_3_, where a slight increase compared to control values was noted. Interestingly, there was no significant difference in *ΦPSII* among different concentrations of the same Ag treatment.

The coefficient of photochemical quenching (*qP*) was not affected upon exposure to AgNP-PVP, since no significant difference compared to control or among treatments with different AgNP-PVP concentrations was observed. On the contrary, AgNP-CTAB, especially 25 and 100 µM concentrations, significantly lowered *qP*, not only compared to control plants but also to those exposed to AgNP-PVP and AgNO_3_, which was particularly pronounced for 25 and 100 µM concentrations. Treatment with 100 µM AgNP-citrate significantly decreased *qP* compared to the control and plants exposed to corresponding concentration of AgNP-PVP- and AgNO_3_, whereas after exposure to 50 µM AgNP-citrate, *qP* was lower only compared to AgNO_3_-treated plants. In the leaves of all AgNO_3_ treated plants, an increase of *qP* values was recorded compared to the control, although it was not statistically significant. Treatments with AgNO_3_ did not induce a significant change in non-photochemical quenching (*NPQ*) compared to the control. Among AgNP treatments, *NPQ* decreased in plants treated with the highest concentration (100 µM) of AgNP-PVP as well as in those treated with all tested concentrations of AgNP-CTAB, which exhibited the strongest negative effect. Interestingly, treatment with 25 µM AgNP-citrate increased *NPQ* values compared to the control and other treatments, whereas the 50 and 100 µM concentrations did not have any significant effect.

### 3.5. Effects on Photosynthetic Pigments

Results of photosynthetic pigment measurements are presented in Table 4. Treatments with AgNPs exhibited stronger effects on total chlorophyll content compared to AgNO_3_. Namely, the 50 and 100 µM AgNP-PVP and AgNP-CTAB treatments significantly decreased the concentrations of total chlorophylls compared to the control as well as to the corresponding concentrations of AgNP-citrate. Among AgNO_3_ treatments, the same effect was recorded only for the highest (100 µM) concentration. The most negative impact was recorded after exposure to 100 µM AgNP-CTAB and AgNO_3_. However, AgNO_3_ treatments had a more negative effect on the chlorophyll *a* to chlorophyll *b* ratio compared to AgNPs since decreased values were recorded upon exposure to all AgNO_3_ concentrations. Among treatments with AgNPs, only 50 µM AgNP-citrate significantly reduced Chl *a/b* values.

Exposures to AgNPs resulted in a stronger impact on carotenoid content in comparison to treatments with AgNO_3_. Namely, the values in leaves of plants treated with all tested AgNP-PVP and AgNP-CTAB concentrations were similarly lower compared to those measured in control plants. However, all treatments with AgNP-citrate resulted in significantly higher carotenoid content than in the control. Among AgNO_3_ treatments, only the highest (100 µM) concentration significantly reduced carotenoid content. Similarly, content of pigments involved in the xanthophyll cycle (VAZ) also decreased in leaves of plants upon exposure to almost all concentrations of AgNP-PVP and AgNP-CTAB, while among AgNO_3_ treatments, only the highest 100 µM concentration exhibited the same effect. However, it may be noted that among treatments with AgNP-citrate, only exposure to 100 µM concentration increased VAZ content.

Carotenoid to chlorophyll ratio (car/chl) was affected only by treatments with AgNPs, among which all concentrations of AgNP-citrate significantly increased the values, while the exposures to all AgNP-PVP treatments and the 25 µM AgNP-CTAB reduced the car/chl ratio compared to the control. 

Results of de-epoxidation state showed that treatments with 25 and 50 µM AgNP-citrate as well as all concentrations of AgNO_3_ increased de-epoxidation, whereas the highest concentrations of AgNP-PVP and AgNP-CTAB decreased it compared to the control and other corresponding treatments.

### 3.6. Effect on Leaf Structure

**Figure 5:**
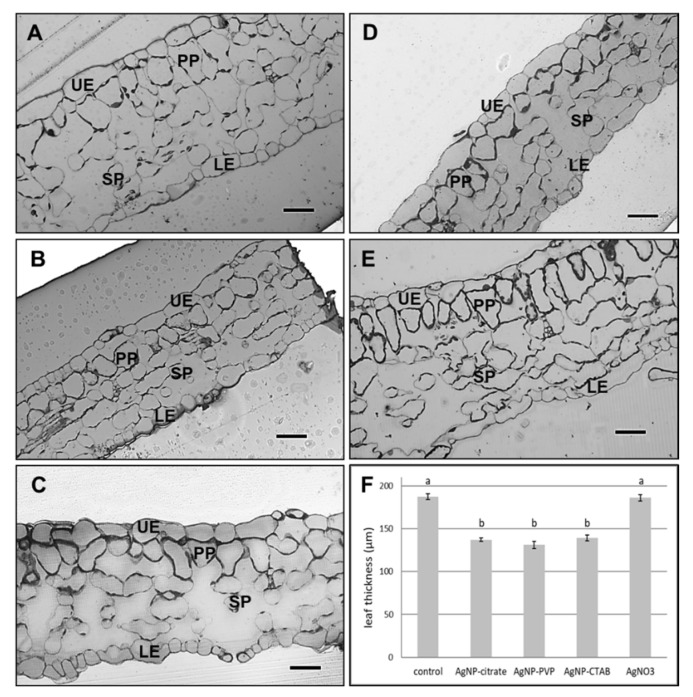
Semithin sections of leaf from (**A**) control plant, and plants exposed to (**B**) 100 µM AgNP-citrate, (**C**) AgNP-PVP, (**D**) AgNP-CTAB and (**E**) AgNO_3_ in liquid ½ strength MS medium as well as (**F**) comparison of leaf semi-thin sections thickness. Bar = 41.8 µm. UE—upper epidermis, LE—lower epidermis, PP—palisade parenchyma, SP—spongy parenchyma. Values are the means ± SE of ten measurements. Columns marked with different letters indicate that treatments are significantly different at the *p* ≤ 0.05 level.

Leaf semithin sections showed changes in the leaf structure and a significant difference in the leaf thickness between plants exposed to 100 µM concentration of all tested AgNPs, as well as to 100 µM AgNO_3_, compared to the control (Figure 5). The leaves of AgNP-exposed plants (Figure 5B–D) were significantly thinner compared to the control (Figure 5A) and AgNO_3_-treated plants (Figure 5E). Furthermore, cells in the spongy parenchyma region in leaves of all treated plants (Figure 5B–E) appeared to be scarce compared to control cells (Figure 5A).

**Figure 6:**
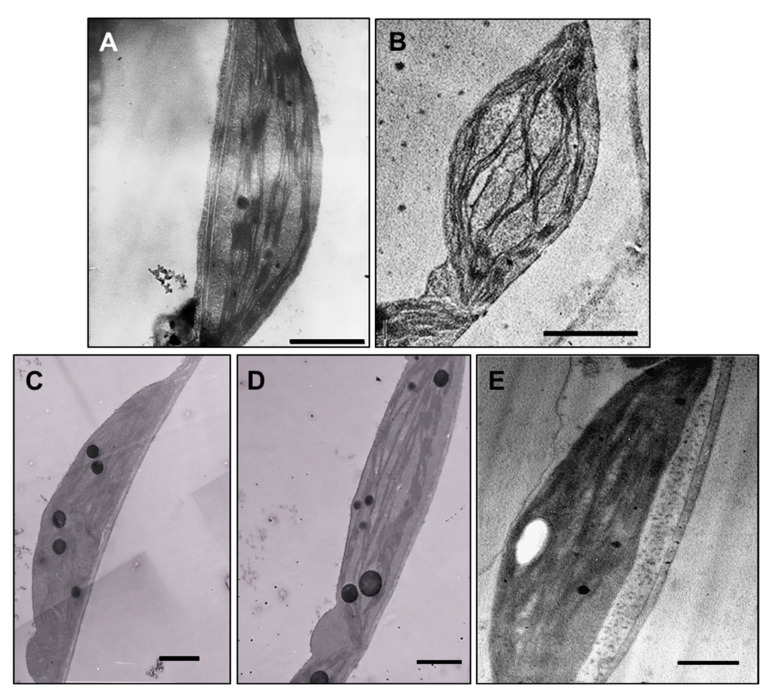
Ultrastructure of leaf chloroplasts from (**A**) control plant (bar = 1 µm), and plants exposed to (**B**) 100 µM AgNP-citrate (bar = 2 µm), (**C**) AgNP-PVP (bar = 1 µm), (**D**) AgNP-CTAB (bar = 1 µm) and (**E**) AgNO_3_ (bar = 1 µm) in liquid ½ strength MS medium.

Ultrastructural studies revealed changes in leaf chloroplasts in all treated plants. The chloroplasts in leaves of plants exposed to 100 µM AgNP-citrate were swollen (Figure 6B), but with no detectable difference in thylakoid system formation compared to the control (Figure 6A). Leaves of AgNP-PVP-treated plants were characterized by thinner and longer chloroplasts with stacked thylakoids and large plastoglobules (Figure 6C) compared to the control. In AgNP-CTAB treated plants, the chloroplasts were long and thin with well-developed thylakoids but with higher content of big plastoglobules compared to the control (Figure 6D). After exposure to AgNO_3_, the least changes in chloroplast ultrastructure were noted; they were the biggest with well-developed thylakoid system and few plastoglobules (Figure 6E). In the leaf tissue, AgNPs were not detected.

## 4. Discussion

Most literature data on AgNP toxicity in various plant species show that AgNPs cause disruption of CO_2_ assimilation efficiency and induce decrease in the photosynthetic pigment content along with reduced chlorophyll fluorescence yield (reviewed in Tkalec et al.) [13]. However, most of these studies investigated only one type of AgNPs (uncoated or with various coatings) without thorough stability analysis during the time of exposure. In the study of Liang et al. [56], in which the effects of AgNPs with different surface coatings were investigated in *Physcomitrella patens*, it was found that AgNP-PVP or AgNP-citrate caused a negligible effect on the chlorophyll of protonemata, whereas AgNPs without surface coating caused much greater damage, suggesting that surface coating alleviated the negative effects of bare AgNPs.

In our study, the effect of the AgNPs on photosynthesis in tobacco leaves was somewhat different, depending on the applied coating. In general, AgNP-PVP and AgNP-CTAB exhibited more negative effects on photosynthetic performance and pigment content than AgNP-citrate. In plants treated with AgNP-citrate, the effective photochemical quantum yield of PSII (*ΦPSII*) and photochemical quenching (*qP*) slightly decreased, implying a reduced electron transport rate, while the nonphotochemical quenching (*NPQ*) value increased. The increase in *NPQ* related to the inhibition of PSII photochemical reactions has also been found in *Arabidopsis* exposed to AgNP-citrate [37,38], *L. gibba* plants treated with uncoated AgNPs [44], and *Vicia faba* exposed to AgNP-PVP [57]. Since AgNPs can reduce the CO_2_ assimilation rate [36,57], and consequently limit demand for NADPH and ATP, increased energy dissipation through *NPQ* and downregulation of electron flow can protect PSII against overexcitation and damage [58]. Furthermore, in AgNP-citrate treated plants we noticed an increase of carotenoids and xanthophylls content as well as elevated car/chl ratio and de-epoxidation state of xanthophylls, pigments that are known to be involved in the *NPQ* of excess energy in the antenna of PSII [59]. Since de-epoxidation of V to A and Z (elevated AZ/VAZ ratio) was found after plant exposure to various plant stresses [60], it can be hypothesized that de-epoxyxanthophylls might quench overexcitation of photosystems induced by AgNP exposure. Carotenoids were also found to play a significant role in antioxidant defence against AgNP-induced ROS in rice [30,61], so it is possible that carotenoids also protected leaves of tobacco plants against ROS, since only a mild oxidative stress was noticed after exposure to 100 µM AgNP-citrate [11]. Furthermore, reduced values of *ΦPSII* and *qP* accompanied by increased carotenoids and xanthophylls were found in tobacco seedlings upon exposure to AgNP-citrate [25], which may imply that AgNP-citrate causes the same response in tobacco plants regardless of the developmental stage.

A severe deterioration of photosynthetic activity was observed after AgNP-CTAB exposure where, besides reduced values of *ΦPSII* and *qP*, a marked decrease in *NPQ* as well as reduction in all photosynthetic pigments were recorded. Several authors reported strong inhibitory effects of AgNPs on PSII photochemistry as a consequence of reduced chlorophyll content [37,38,43,44,62], either through destruction of photosynthetic pigments or inhibited chlorophyll biosynthesis [36]. Decreased *NPQ* values accompanied with reduced carotenoids content and lower de-epoxidation state of xanthophylls observed in tobacco plants upon exposure to AgNP-CTAB and AgNP-PVP can be related with the decreased content of chlorophylls. Significant decrease in total chlorophyll content may reduce efficiency of light absorption and subsequent conversion of light energy into electron transport, which can ultimately decrease the photosynthesis efficiency and eliminate the need for energy dissipation. In the aquatic plant *S. polyrhiza*, AgNP-PVP inhibited the photoprotective capacity of PSII and decreased carotenoid content [62].

The difference in effects of various AgNPs on photosynthesis in tobacco plants could be correlated with their distinct behavior in the exposure medium (dependent on the applied coating), which can modify the initial properties of AgNPs and influence their bioavailability and uptake [13]. Ag uptake was significantly higher in leaf cells of plants exposed to PVP- and CTAB-coated AgNPs compared to values obtained after exposure to AgNP-citrate. Fast agglomeration of AgNP-citrate observed in the liquid medium with high ionic strength could have reduced their availability and prevented their efficient uptake by root cells. Conversely, agglomeration proceeded at a slower rate for AgNP-PVP compared to AgNP-citrate, which indicates that they were available for uptake for a longer period. Interestingly, AgNP-CTAB also agglomerated rapidly, but after 48 h re-stabilization of small agglomerates was observed. Rapid agglomeration of commercial AgNP-citrate in liquid ½ strength MS medium was also reported by [25]. In contrast, in a proteomic study of tobacco plants exposed to AgNP-citrate in ultrapure water, AgNPs proved to be very stable and thus more available for uptake, causing a severe impact on photosynthesis by down-regulating photosynthesis-related proteins [63]. As for the CTAB-coated AgNPs, there is also a possibility that their more toxic effects on photosynthesis are correlated with their surface charge. Stability analyses showed that AgNP-CTAB kept their positive charge in a liquid ½ strength MS medium; on the contrary, the negative ζ potential of AgNP-citrate decreased slightly, while the ζ potential became even more negative with time for AgNP-PVP. Studies have already shown that positively charged AgNP-CTAB had a more severe impact on plant growth [19] and appearance of oxidative stress [18] than negatively charged citrate or non-ionic PVP coatings, probably due to attachment of positively charged AgNPs to the negatively charged plant cell walls. Significantly higher toxicity of positively charged AgNP-cystamine compared to AgNP-citrate was also reported for wheat calli [64]. All of the above confirms the importance of AgNP stability analysis in the exposure medium in determining AgNP effects. However, it is still insufficiently explored what happens to the AgNPs after they enter plant cells. In our previous investigations on tobacco plants exposed to AgNP-citrate, the direct AgNP uptake by root cells was confirmed by TEM and EDX analyses, although no AgNPs were detected in the leaves of exposed plants [11]. In this study we also could not detect AgNPs in leaf tissue. So far, detection of AgNPs in leaves showed ambiguous results and, in addition to nanoparticles, a mixture of AgNPs and secondary species, including Ag-thiol, and other Ag^+^ species have been detected [65]. The mechanisms of AgNPs internalization in roots as well as upward transport to shoots are still insufficiently known (reviewed in [13].

It cannot be ruled out that conditions during exposure did not result in the oxidation of AgNPs and subsequent release of Ag^+^ ions [66], which after translocation to the plants caused the observed effects. It is known that Ag^+^ ions can bind to plastocyanin replacing Cu^+^ ions, resulting in disturbed photosynthetic electron transport [67]. To discriminate between the effects of Ag in the form of nanoparticles and ions, we also analyzed leaves of plants exposed to AgNO_3_ and found that this treatment was less toxic than those with either of the AgNPs. Exposure to AgNO_3_ had no significant effects on fluorescence parameters in any of the tested concentrations compared to the control. Moreover, the *ΦPSII* and *qP* values were significantly higher upon exposure to AgNO_3_ compared to the corresponding concentrations of AgNP-citrate and AgNP-CTAB. As for the photosynthetic pigments, a significant decrease was found for total chlorophylls, carotenoids and xanthophylls at the highest applied concentration (100 µM) of AgNO_3_; however, this decrease was less pronounced compared to exposure to 100 µM AgNP-CTAB. In the study of Ke et al. [38], both AgNP-citrate and Ag^+^ ions applied in the same concentrations affected the photosynthesis efficiency in *A. thaliana* leaves, but AgNPs induced more severe inhibitory effects, which corroborates our results. On the other hand, impact of AgNO_3_ on pigment content and *F_v_/F_m_* in *Brassica* seedlings was stronger than that of AgNPs [34]. Similarly, in tobacco seedlings, AgNO_3_ had a more negative effect on relative electron transport rate and *qP* than the corresponding treatments with AgNP-citrate, while the content of photosynthetic pigments increased after treatment with these nanoparticles [25]. This discrepancy in results with respect to the current study can be correlated with the plant developmental stage; it could be that seedlings, given the increased need for nutrients, are more susceptible to AgNO_3_-imposed stress compared to fully developed adult plants.

Microscopy analyses confirmed that all AgNPs had more negative effects on tobacco leaf (ultra)structure than AgNO_3_. Namely, leaves of all AgNP-exposed plants were significantly thinner compared to the control and AgNO_3_-treated plants. In our previous study, thinner leaves were observed after exposure of adult tobacco plants to 100 µM concentration of commercial AgNP-citrate in ultrapure water [11]. Moreover, changes in size and shape of mesophyll cells after AgNP exposure have been noted in bean [68] and pea seedlings [35]. Although no AgNPs were detected in the leaves of plants exposed to either type of AgNPs, treatment with 100 μM AgNPs induced alterations in chloroplasts and plastoglobules, mainly in shape and size. Contrary to AgNO_3_-treated plants, which revealed large chloroplasts with a few plastoglobules, thin and long chloroplasts with a greater amount of large plastoglobules were noticed in leaves upon exposure to AgNP-PVP and AgNP-CTAB. Plastoglobules are subcompartments of thylakoids, containing enzymes that participate in lipid metabolic pathways. It is well documented that under biotic and abiotic stress conditions, the size and the number of plastoglobules increase [69,70], and they may be derived from thylakoid disassembly [71] as well as from the degradation of chlorophyll, carotenoids and photosynthetic proteins [72]. Apart from the impact on size of chloroplasts and plastoglobules, disturbances in thylakoid system in the form of stacked thylakoids were observed in the leaves of plants exposed to 100 µM AgNP-PVP. A dilated or disrupted thylakoid system has been already observed after treatment with AgNPs [12,15,48]. Moreover, in studies on duckweed, damage to the thylakoid system was found after exposure to the AgNPs along with a decline in the photosynthetic pigments, demonstrating that AgNPs considerably impaired the structural and functional integrity of the chloroplasts [73,74], which corresponds to our results.

## 5. Conclusions

Coating-dependent effects of investigated AgNPs on photosynthetic performance and chloroplast ultrastructure were recorded. AgNP-citrate induced only mild effects on chlorophyll *a* fluorescence parameters and pigment content, which can be correlated with their fast agglomeration in the exposure medium and therefore weak uptake. Treatments with AgNP-PVP and AgNP-CTAB exhibited more severe effects on tobacco photosynthesis, probably as a result of higher stability and Ag accumulation. However, there is also a possibility that the observed effects of CTAB-coated AgNPs are also correlated with their positive surface charge. The least impact on tobacco photosynthesis and pigment content was recorded upon AgNO_3_ exposure, thus indicating that the toxic effects of AgNPs cannot be ascribed only to release of Ag^+^ ions.

## Figures and Tables

**Table 1:** Physicochemical characteristics of AgNP-citrate, AgNP-PVP, and AgNP-CTAB in ultrapure water by means of hydrodynamic diameter (*d*_H_) obtained from size distributions by volume, polydispersity index, ζ potential, surface plasmon resonance (SPR) peak wavelength, and percentage of ionic Ag^+^.

Characteristics		AgNP-citrate	AgNP-PVP	AgNP-CTAB
Size peak I	*d*_H_, nm mean volume, %	5.99 ± 1.25 32.4%	57.96 ± 17.54 100%	28.26 ± 10.28 18.1%
Size peak II	*d*_H_, nm mean volume, %	24.03 ± 8.14 67.6%		55.99 ± 11.60 23.9%
Size peak III	d*_H_*, nm mean volume, %			163.30 ± 85.96 58.0%
Polydispersity index		0.327	0.473	0.545
ζ potential, mV		−23.78 ± 1.17	−4.24 ± 2.57	44.67 ± 3.36
SPR peak, nm		420	465	410
Ag^+^, %		0.4	0.3	0.5
Working stock concentrations, mM		10.5	9.8	11.5

**Table 2:** Temporal evolution of zeta (ζ) potentials and aggregates of 100 µM AgNP-citrate, AgNP-PVP, and AgNP-CTAB after being added to exposure solution (liquid ½ strength MS medium). Values are the means ± SE of six measurements.

	AgNP-Citrate		AgNP-PVP		AgNP-CTAB	
Time	Zeta Potential (mV)	Diameter (nm)	Zeta Potential (mV)	Diameter (nm)	Zeta Potential (mV)	Diameter (nm)
10 min	−20.8 ± 2.26	213.6 ± 123.5	−9.63 ± 0.91	124.3 ± 92.9	16.50 ± 3.49	468.9 ± 143.6
1 h	−22.72 ± 0.95	422.6 ± 136.6	−12.00 ± 0.70	152.8 ± 64.2	7.00 ± 0.91	510.7 ± 102.2
4 h	−23.05 ± 0.46	407.8 ± 125.8	−13.13 ± 1.40	317.5 ± 153.4	4.95 ± 1.40	408.2 ± 138.7
24 h	−22.18 ± 0.80	339.5 ± 97.4	−11.97 ± 1.27	448.7 ± 132.7	11.12 ± 1.75	398.0 ± 144.7
48 h	−19.78 ± 1.36	245.1 ± 46.5	−11.07 ± 1.14	450.6 ± 128.0	9.31 ± 2.20	101.6 ± 13.5 393.4 ± 107.6

**Table 3:** Content of Ag and chlorophyll fluorescence parameters (expressed as % of control) in leaves after 7 days of tobacco plants exposure to 0, 25, 50, and 100 µM AgNP-citrate, AgNP-PVP, AgNP-CTAB, and AgNO_3_ in a liquid ½ strength MS medium. *F_v_*/*F_m_*—maximum fluorescence in dark-adapted state, *ΦPSII*—effective quantum yield of PSII, *qP*—coefficient of photochemical quenching, *NPQ*—nonphotochemical quenching. Values are the means ± standard error of three different experiments, each with three replicates. Columns marked with different letters indicate that treatments are significantly different at the *p* ≤ 0.05 levels (LSD test); small letters mark the differences among AgNPs or AgNO_3_ concentrations, capital letters mark the differences among AgNPs or AgNO_3_ of the same concentration, whereas asterisks (*) mark the differences between each treatment and control. #—Ag content was below the limit of quantification (<0.1 µg g^−1^).

Treatments	AgNP or AgNO_3_ (µM)	Ag Content in Leaves (µg/g_DW_)	*F_v_*/*F_m_*	*ΦPSII*	*qP*	*NPQ*
Control	0	0 #	100 ± 0.43	100 ± 10.85	100 ± 9.57	100 ± 9.23
	25	0.49 ± 0.1 ^a,B^	100 ± 0.24	80.95 ± 9.61 ^BC^	92.23 ± 6.93 ^A^	126.86 ± 10.64 *^,a,A^
AgNP-citrate	50	0.62 ± 0.15 ^a,D^	100.51 ± 0.31 ^A^	83.33 ± 16.67 ^B^	83.82 ± 13.33 ^B^	107.33 ± 9.98 ^ab,A^
	100	3.65 ± 0.9 *^,b,B^	100.34 ± 0.41	79.37 ± 11.74 ^B^	72.81 ± 10.35 *^,B^	95.43 ± 8.21 ^b, A^
	25	0.64 ± 0.12 ^a,B^	99.62 ± 0.36 ^b^	93.51 ± 10.99 ^AB^	92.32 ± 9.56 ^A^	94.20 ± 7.13 ^a,B^
AgNP-PVP	50	5.25 ± 0.28 *^,b,A^	100.62 ± 0.20 ^a,A^	101.45 ± 5.75 ^AB^	98.62 ± 4.85 ^AB^	96.48 ± 4.46 ^a,A^
	100	7.47 ± 0.65 *^,b,AB^	100.65 ± 0.24 ^a^	108.70 ± 8.51 ^A^	99.16 ± 6.91 ^A^	76.68 ± 5.13 *^,b,BC^
	25	2.11 ± 0.62 *^,a,A^	100.46 ± 0.22 ^ab^	60.71 ± 7.65 *^,C^	65.02 ± 7.79 *^,ab,B^	63.39 ± 7.88 *^,C^
AgNP-CTAB	50	3.4 ± 0.25 *^,a,B^	99.43 ± 0.41 ^b,B^	78.57 ± 12.04 ^B^	82.95 ± 10.49 ^a,B^	69.12 ± 8.06 *^,B^
	100	8.84 ± 1.33 *^,b,A^	100.8 ± 0.32 ^a^	57.29 ± 8.40 *^,B^	56.25 ± 8.42 *^,b,B^	59.54 ± 6.33 *^,C^
	25	0.91 ± 0.05 ^a,B^	99.65 ± 0.45	114.94 ± 12.75 ^A^	115.69 ± 10.12 ^A^	100.97 ± 8.99 ^B^
AgNO_3_	50	1.40 ± 0.06 *^,b,C^	100 ± 0.35 ^AB^	133.33 ± 16.55 ^A^	122.87 ± 13.99 ^A^	94.19 ± 11.47 ^A^
	100	5.3 ± 0.29 *^,c,B^	100.17 ± 0.17	128.74 ± 8.95 ^A^	113.07 ± 7.58 ^A^	88.71 ± 5.82 ^AB^

**Table 4:** Contents of photosynthetic pigments and their ratios (expressed as % of control) in leaves after 7 days of tobacco plants exposure to 0, 25, 50, and 100 µM AgNP-citrate, AgNP-PVP, AgNP-CTAB, and AgNO in a liquid ½ strength MS medium. VAZ—violaxanthin, antheraxanthin and zeaxanthin (xanthophyll cycle pool), chl *a*/*b*—chlorophyll *a*/*b*, car/chl—carotenoids/chlorophyll, (AZ)/(VAZ)—de-epoxidation state of xanthophylls. Values are the means ± standard error of three different experiments, each with three replicates. Columns marked with different letters indicate that treatments are significantly different at the *p* ≤ 0.05 levels (LSD test); capital letters mark the differences among AgNP concentrations as well as control, small letters mark the differences among AgNO_3_ concentrations as well as control, while asterisks (*) mark the differences among different treatments of the same concentration.

Treatments	AgNP or AgNO_3_ (µM)	Chlorophylls	Carotenoids	VAZ	chl *a*/*b*	car/chl	(AZ)/(VAZ)
		as % of control					
Control	0	100.00 ± 3.10	100.00 ± 5.17	100.00 ± 3.80	100.00 ± 2.54	100.00 ± 2.16	100.00 ± 4.78
	25	104.22 ± 2.15 ^A^	111.53 ± 3.69 *^,a,A^	99.30 ± 2.36 ^b,AB^	99.01 ± 0.34 ^a,A^	107.11 ± 1.96 *^,A^	113.30 ± 2.59 *^,b,A^
AgNP-citrate	50	97.99 ± 2.04 ^B^	102.18 ± 2.65 ^b,A^	92.78 ± 1.26 ^b^	93.20 ± 1.49 *^,b,B^	104.39 ± 0.80 *^,A^	130.85 ± 2.14 *^,a,A^
	100	104.95 ± 1.53 ^A^	109.42 ± 1.51*^,ab,A^	115.85 ± 1.30 *^,a,A^	102.64 ± 0.32 ^a,A^	104.41 ± 1.19 *^,A^	94.10 ± 3.92 ^c,B^
	25	95.86 ± 2.39 ^B^	86.89 ± 5.23 *^,B^	87.45 ± 1.57 *^,C^	98.03 ± 0.70 ^A^	90.57 ± 3.25 *^,B^	94.55 ± 2.25 ^ab,C^
AgNP-PVP	50	90.41 ± 1.43 *^,C^	80.74 ± 3.15 *^,B^	88.38 ± 1.17 *	100.96 ± 0.50 ^A^	89.51 ± 4.60 *^,C^	102.77 ± 2.80 ^a,C^
	100	91.06 ± 0.61 *^,AB^	82.96 ± 2.89 *^,B^	89.17 ± 1.90 *^,B^	98.56 ± 0.07 ^B^	91.17 ± 2.89 *^,B^	89.34 ± 1.60 *^,b,BC^
	25	98.22 ± 2.62 ^a,AB^	88.07 ± 3.60 *^,a,B^	92.09 ± 5.05 ^a,BC^	99.43 ± 0.76 ^A^	89.58 ± 1.25 *^,a,B^	100.48 ± 3.47 ^a,BC^
AgNP-CTAB	50	91.55 ± 2.97 *^,b,C^	93.43 ± 4.48 *^,a,A^	88.78 ± 2.57 *^,b^	99.25 ± 0.57 ^A^	103.39 ± 1.77 ^b,AB^	105.58 ± 1.62 ^a,C^
	100	77.82 ± 3.68 *^,c,B^	78.68 ± 4.38 *^,b,B^	78.60 ± 4.09 *^,b,B^	97.85 ± 2.14 ^BC^	101.02 ± 1.27 ^b,A^	85.04 ± 0.45 *^,b,C^
	25	103.54 ± 2.01 ^a,A^	106.76 ± 6.84 ^a,A^	109.23 ± 3.45 ^a,A^	92.82 ± 0.64 *^,B^	102.94 ± 4.71 ^A^	109.50 ± 3.15 *^,AB^
AgNO_3_	50	104.77 ± 1.42 ^a,A^	101.60 ± 1.76 ^a,A^	96.89 ± 1.39 ^a^	90.69 ± 0.58 *^,B^	97.02 ± 1.92 ^BC^	116.40 ± 1.28 *^,B^
	100	86.15 ± 7.58 *^,b,B^	85.97 ± 10.04 *^,b,B^	83.37 ± 7.93 *^,b,B^	94.50 ± 0.35 *^,C^	99.27 ± 3.56 ^A^	109.97 ± 2.15 *^,A^

## Data Availability

The data used to support the findings of this study are included within the article.

## Supplementary Materials

The following are available online at https://www.mdpi.com/2079-4991/11/3/744/s1, Figure S1: Size distribution diagrams obtained from TEM images of stock solutions of AgNPs coated with (A) citrate (AgNP-citrate), (B) polyvinylpyrrolidone (PVP; AgNP-PVP) and (C) cetyltrimethylammonium bromide (CTAB; AgNP-CTAB) in ultrapure water. Figure S2: UV-Vis absorption spectra of 100 µM AgNPs in a liquid ½ strength MS medium recorded over a period of 48 hours; (A) AgNP-citrate, (B) AgNP-PVP and (C) AgNP-CTAB. Insets show AgNP agglomeration recorded after 4 h by TEM analyses.
