# Supplementary material for: Phytotoxicity of Silver Nanoparticles on Tobacco Plants: Evaluation of Coating Effects on Photosynthetic Performance and Chloroplast Ultrastructure
Source: Nanomaterials (Basel). 2021 Mar 16;11(3):744. doi: 10.3390/nano11030744 (PMC8002358; doi:10.3390/nano11030744)
Supplement: Supplementary file 1 [file nanomaterials-11-00744-s001.pdf]

Article

Phytotoxicity of Silver Nanoparticles on Tobacco Plants: Evaluation of Coating Effects on Photosynthetic Performance and Chloroplast Ultrastructure

Petra Peharec Štefanić [1], Karla Košpić [1], Daniel Mark Lyons [2], Lara Jurković [2], Biljana Balen [1] and Mirta Tkalec [1,*]

[1] Department of Biology, Faculty of Science, University of Zagreb, Horvatovac 102a, HR-10000 Zagreb, Croatia; ppeharec@biol.pmf.hr (P.P.Š.); karla.kospic@biol.pmf.hr (K.K.); bbalen@biol.pmf.hr (B.B.)
[2] Center for Marine Research, Ruđer Bošković Institute, G. Paliaga 5, 52210 Rovinj, Croatia; Daniel.Mark.Lyons@irb.hr (D.M.L.); Lara.Jurkovic@irb.hr (L.J.)
* Correspondence: mtkalec@biol.pmf.hr

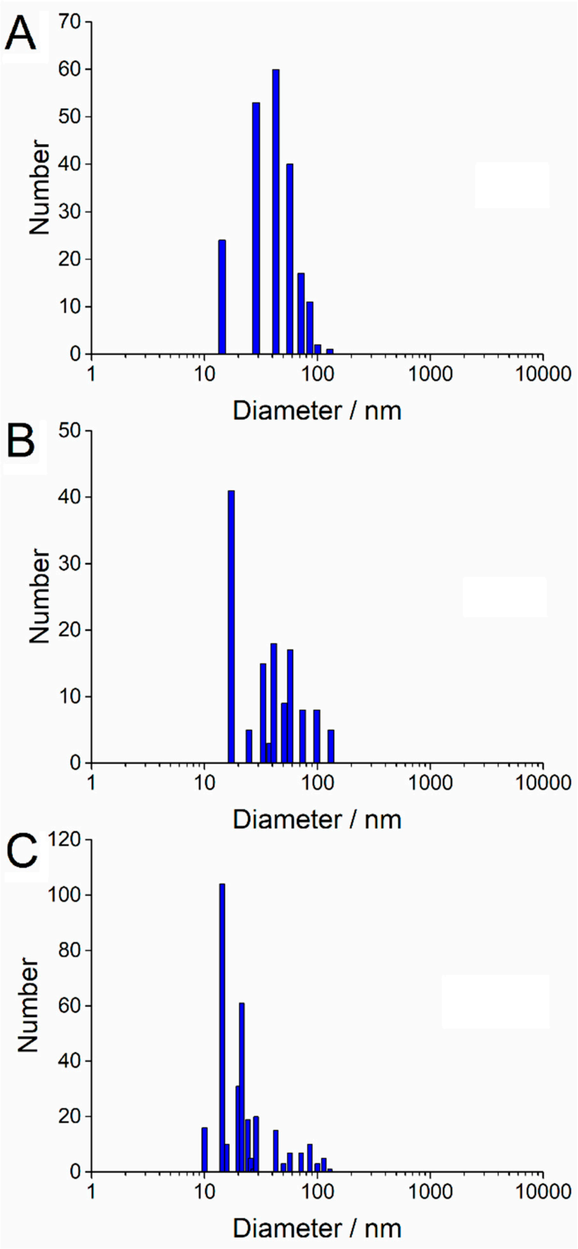

Figure S1. Size distribution diagrams obtained from TEM images of stock solutions of AgNPs coated with A) citrate (AgNP-citrate), B) polyvinylpyrrolidone (PVP; AgNP-PVP) and C) cetyltrimethylammonium bromide (CTAB; AgNP-CTAB) in ultrapure water.

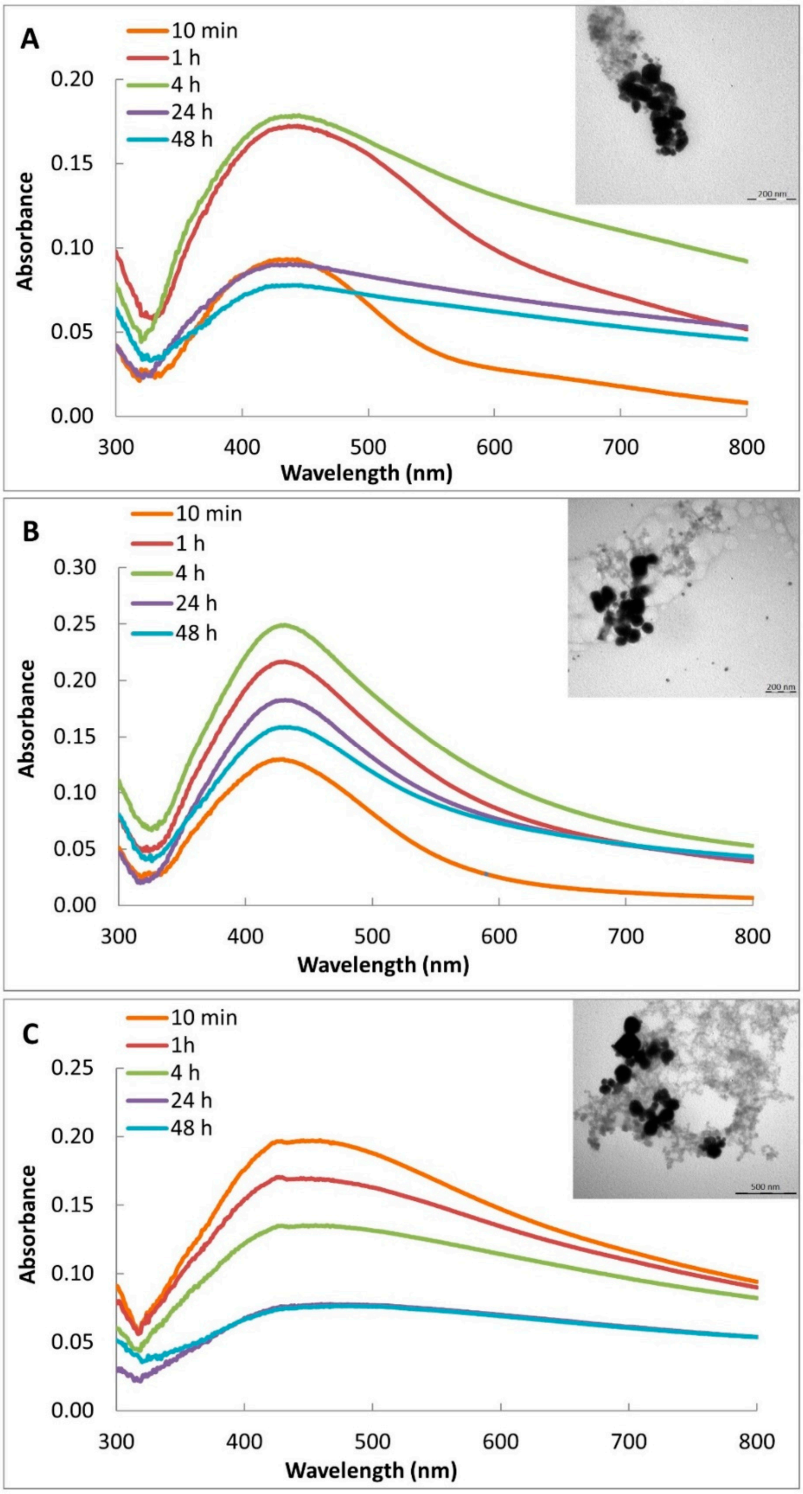

Figure S2. UV-Vis absorption spectra of 100 µM AgNPs in a liquid ½ strength MS medium recorded over a period of 48 hours; (A) AgNP-citrate, (B) AgNP-PVP and (C) AgNP-CTAB. Insets show AgNP agglomeration recorded after 4 h by TEM analyses.
